# Salivary gland derived peptides as a new class of anti-inflammatory agents: review of preclinical pharmacology of C-terminal peptides of SMR1 protein

**DOI:** 10.1186/1476-9255-7-49

**Published:** 2010-09-28

**Authors:** Ronald D Mathison, Joseph S Davison, A Dean Befus, Daniel A Gingerich

**Affiliations:** 1 Faculty of Medicine, University of Calgary, 3330 Hospital Drive NW, Calgary, Alberta, T2N 4N1, Canada; 2 550A Heritage Medical Research Centre, Faculty of Medicine and Dentistry, University of Alberta, Edmonton, Alberta, T6G 2S2, Canada; 3 Turtle Creek Biostatistical Consulting, 2219 Wilmington Road, Lebanon, OH 45036, USA

## Abstract

The limitations of steroidal and non steroidal anti-inflammatory drugs have prompted investigation into other biologically based therapeutics, and identification of immune selective anti-inflammatory agents of salivary origin. The traditional view of salivary glands as accessory digestive structures is changing as their importance as sources of systemically active immunoregulatory and anti-inflammatory factors is recognized. Salivary gland involvement in maintenance of whole body homeostasis is regulated by the nervous system and thus constitutes a "neuroendocrine axis". The potent anti-inflammatory activities, both *in vivo *and *in vitro*, of the tripeptide Phe-Glu-Gly (FEG) are reviewed. FEG is a carboxyl terminal peptide of the prohormone SMR1 identified in the rat submandibular salivary gland, The D-isomeric form (feG) mimics the activity of its L-isomer FEG. Macropharmacologically, feG attenuates the cardiovascular and inflammatory effects of endotoxemia and anaphylaxis, by inhibition of hypotension, leukocyte migration, vascular leak, and disruption of pulmonary function and intestinal motility. Mechanistically, feG affects activated inflammatory cells, especially neutrophils, by regulating integrins and inhibiting intracellular production of reactive oxygen species. Pharmacodynamically, feG is active at low doses (100 μg/kg) and has a long (9-12 hour) biological half life. As a therapeutic agent, feG shows promise in diseases characterized by over exuberant inflammatory responses such as systemic inflammatory response syndrome and other acute inflammatory diseases. Arthritis, sepsis, acute pancreatitis, asthma, acute respiratory inflammation, inflammatory bowel disease, and equine laminitis are potential targets for this promising therapeutic peptide. The term "Immune Selective Anti-Inflammatory Derivatives" (ImSAIDs) is proposed for salivary-derived peptides to distinguish this class of agents from corticosteroids and nonsteroidal anti-inflammatory drugs.

## Introduction

Saliva, best known for its digestive and protective properties in the maintenance of the health and integrity of the oral and gastric mucosa [1], is becoming increasingly recognized for its important role in regulating whole body homeostasis [2]. Although over the past half century many bioactive proteins and peptides have been identified in saliva [3,4], salivary glands are still viewed primarily as accessory digestive structures that provide lubrication and digestive enzymes. However, it is now becoming clear that salivary endocrine factors play an important role in the modulation of systemic immune and inflammatory reactions. Classically, the salivary glands are generally considered as exocrine glands that dispense their protein and fluid externally into a lumen or a duct. However, investigations dating from 60 years ago suggested an unorthodox view that salivary and other exocrine glands, such as the pancreas, are capable of endocrine secretion, dispensing their secretions internally, i.e. directly into the blood stream. It has been suggested that these glands be called "duacrine" glands [5].

Salivary glands produce various immunoregulatory [6,7] and anti-inflammatory [8] agents. The importance of the salivary gland in maintaining homeostasis has been clarified in recent decades by demonstration of neuroendocrine interactions between the nervous, endocrine, and immune systems [9]. The salivary glands, as well as the thymus and cervical lymph nodes, are innervated by noradrenergic fibers from the sympathetic trunk [10,11], which were shown to modulate lymphocyte function within lymph nodes and thymus [12,13].

This paper reviews the published pharmacologic and immunopharmacologic evidence that salivary gland derived peptides, with particular emphasis on the D-isomeric tripeptide feG, deserve consideration as potentially therapeutically useful anti-inflammatory agents.

## The Neuroendocrine Axis

**Figure 1:**
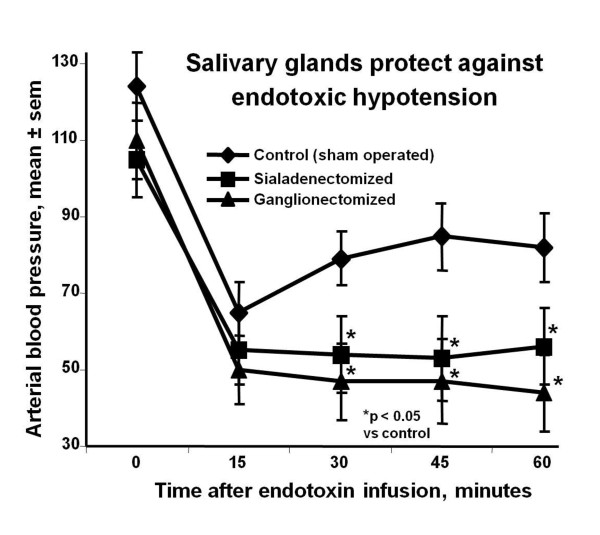
**Neuroendocrine axis and modulation of responses to lipopolysaccharide**: Intravenous administration of lipopolysaccharide (LPS) induces rapid reduction in blood pressure in rats. Either bilateral removal of the submandibular salivary glands (sialadenectomized) or the superior cervical ganglia (ganglionectomized) exacerbate the LPS-induced hypotension. (mean ± sem, n = 6 to 8). Adapted from [16].

The existence of salivary-derived, systemically acting, anti-inflammatory factors and the regulation of salivary gland function by the sympathetic nervous system were demonstrated in anaphylaxis and endotoxemia models in rats. Superior cervical ganglionectomy significantly reduced mortality and greatly attenuated the influx of histamine, neutrophils, and serum-derived proteins, into bronchoalveolar fluid in anaphylaxis-induced pulmonary inflammation in rats [14]. However, the protective effect of superior cervical ganglionectomy was completely abolished in rats with concurrent bilateral sialadenectomy of the submandibular salivary glands [15]. These findings reveal that submandibular salivary glands produce systemically important immunomodulatory factors and that the cervical sympathetic nerves tonically inhibit the release of some of these factors. In an endotoxin-induced acute hypotension model, either bilateral superior cervical ganglionectomy or submandibular sialadenectomy resulted in significantly larger drops in blood pressure compared to intact controls [16] (Figure 1). These results indicate that the submandibular gland elaborates factors that protect against acute hypotension induced by endotoxin and that these factors are under the control of the cervical sympathetic nervous system.

## Bioactivity of Salivary Gland Extracts: SGP-T

On the basis of the findings that salivary glands participate in modulating systemic inflammatory responses, bioactive factors were sought in saliva. Extracts of submandibular glands were subjected to molecular weight cut-off filtration and tested for bioactivity. A novel seven amino acid peptide with sequence Thr-Asp-Ile-Phe-Glu-Gly (TDIFEGG) was isolated, named submandibular peptide-T (SGP-T), and shown to express anti-allergic and anti-endotoxin activities[16,17]. SGP-T was identified as the carboxyl terminal of SMR1, a 146-amino acid, multipotent prohormone product of the VCSa1 (variable coding sequence A1) gene [18], which is also identified as RATSMR1A, Smr1, SMR1 protein and VCS-alpha 1. Recent studies have shown that SMR1 is secreted into saliva in response to intraperitoneal administration of β-adrenergic and cholinergic agonists, and removal of the cervical sympathetic ganglia that innervate the salivary glands resulted in increased levels of SMR1 protein in the submandibular glands [19]. These observations are in keeping with a cervical sympathetic trunk - submandibular gland axis propounded previously [15].

**Figure 2:**
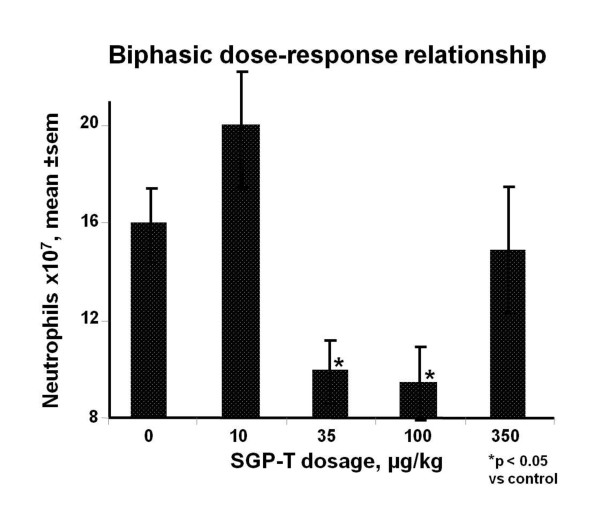
**SGP-T and neutrophil chemotaxis**: Neutrophil chemotaxis into carrageenan-soaked sponges over a 24 hour period in rats is inhibited by SGP-T injected intravenously, in a bell-shaped dose-dependent manner, at dosages indicated. (mean ± sem, n = 3 to 12). Adapted from [22].

In ovalbumin (OA) sensitized rats SGP-T at dosages of 35 and 100 μg/kg injected 10 minutes prior to OA challenge protected against anaphylactic hypotension [20]. Interestingly, neither lower nor higher doses (10 or 350 μg/kg) of SGP-T were protective. In OA sensitized rats challenged intra-intestinally with OA, pretreatment with SGP-T dose-dependently reduced the incidence and duration of disrupted intestinal motility and prevented the development of diarrhea [20]. SGP-T treatment also significantly suppressed endotoxin-induced fever in rats [21]. Neutrophil migration into carrageenan soaked sponges was inhibited by SGP-T injected intravenously at 100 μg/kg at -1, 0, or 4 hours after implantation [22]. Interestingly, dose-response assays showed a bell-shaped dose response curve; neither lower (10 μg/kg) or higher (350 μg/kg) inhibited neutrophil migration (Figure 2). SGP-T treatment also promoted a bell-shaped dose-dependent recovery in the ability of neutrophils obtained from carrageenan-soaked sponges to generate superoxide anion. In another study endotoxin-induced leukocyte rolling and adhesion, quantified *in vivo *by intravital microscopy of mesenteric venules in anesthetized rats, was prevented by pre-treatment with SGP-T [23].

Before considering the pharmacology of SGP-T and its analogues a brief summary of the VCSa1 gene family and its products is presented as this subject was recently reviewed [24].

## The VCSa1 Gene Family

**Figure 3:**
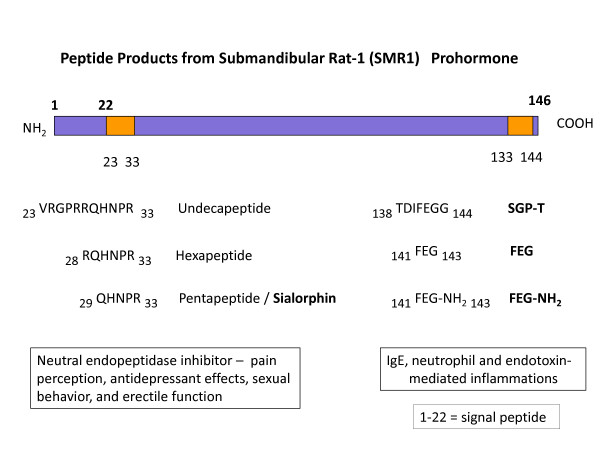
**eptide Products from Submandibular Rat-1 (SMR1) Prohormone**: The SMR1 precursor protein contains sialorphin near the N-terminal, and SGP-T (submandibular gland peptide T) near the C-terminal. FEG and FEG(NH_2_) are biologically active derivatives of SGP-T.

The *Vcsa1 *gene that encodes the rat SMR1 protein is a member of the variable coding sequence multigene family, which share a common gene structure but exhibit extensive sequence variation in the coding region of the genes [25]. The VCS genes, which are divided into two subgroups VCSA and VCSB, are found exclusively in mammals [26]. The VCSA family, containing the Vcsa1 gene, has emerged recently, and exclusively in rodents, whereas the proline-rich VCSB family is found in all placental mammals [27]. Human members of the VCSB family include *PROL1*, *SMR3B (PROL3)*, and *SMR3A (PROL5) *[24], *and encode *salivary and lacrimal secreted proline-rich proteins [28-30]. The SMR1 protein product of the rat *Vcsa1 *gene is cleaved into at least two biologically active peptides, sialorphin (QHNPR) and SGP-T (TDIFEGG) (Figure 3). Whereas the N-terminal QHNPR sequence is conserved in all products of the rat VCSA family members, the C-terminal TDIFEGG sequence is absent due to mutation or truncation of the C-terminus [27]. With the absence of the VCSA subgroup of genes in non-rodent mammals, sialorphin and SGP-T may not be present, although homologues of these peptides are encoded by VCSB genes. The human VCSB gene *PROL1 *encodes a protein that contains a QRFSR motif (opiorphin) that is functionally equivalent to rat sialorphin [31], although a homologue of TDIFEGG (SGP-T) has not been identified yet. Sialorphin participates in diverse physiological processes, such as pain perception, antidepressant effects, sexual behavior, and erectile function [4,32-34], and these actions appear to be related the inhibition of neutral endopeptidase (NEP)[4]. Human opiorphin has similar activity [35]. Vcsa1 expression is hormonally regulated by androgens [33,36], and the expression of opiorphin family genes may be similarly regulated [37].

## Pharmacology of the Tripeptide D-PHE-D-GLU-GLY (feG)

During SGP-T isolation and testing procedures, the truncated sequence Phe-Glu-Gly (FEG) was identified, which itself showed bioactivity, as did its D-isomeric form (feG) [17]. This tripeptide sequence was synthesized and characterized pharmacologically in various models.

### Animal Models

Several rat models of systemic inflammatory disease, and *in vitro or ex vivo *immunopharmacologic assays were utilized to test the bioactivity of feG as follows.

• Endotoxemia models. Injection of lipopolysaccharide (LPS) in rats results in rapid transient decreases in blood pressure, increases in circulating leukocytes, migration of leukocytes into peritoneal fluid, accumulation of neutrophils in cardiac tissue, disrupted intrinsic rhythmicity of migrating myoelectric complexes (MMC) in intestines, etc.

• Anaphylaxis. Rats sensitized to ovalbumin (OA) or larvae of *Nippostrongylis braziliensis *(Nb) and subsequently challenged with these same antigens by injection, orally, or intra-nasally depending on the purposes of the experiment, develop rapid drops in blood pressure; accumulation of leukocytes in cardiac tissue; increases in vascular permeability; increased circulating leukocytes; diarrhea and disrupted MMCs; and IgE-mediated migration of eosinophils, neutrophils, and monocytes into airways.

• Pulmonary bronchoconstriction (measured by specific lung resistance) and airway hyper-responsiveness to methacholine or carbachol in sheep naturally allergic to *Ascaris suum *or in rats sensitized with either OA or with larvae of Nb and challenged by aerosol administration of the sensitizing antigens was measured after aerosol challenge with the antigen.

• Spinal cord injury in rats induced by 60 second clip compression of the spinal cord was measured by lesion site histology and histochemistry as well as recovery of locomotor function.

• Pancreatitis induced in mice by intravenous injection of caerulein was measured histologically, by determination of plasma amylase and lipase activity, and by immunoassays.

• *In vitro *and *ex vivo *studies on leukocyte migration, adhesion, cell surface marker expression, and reactive oxygen species production.

#### Hypotension

**Figure 4:**
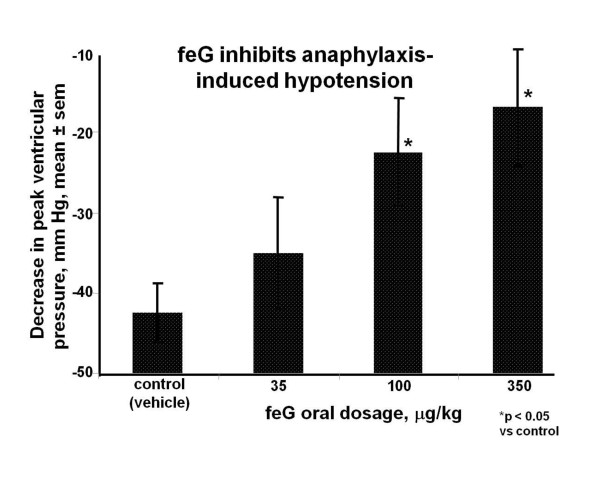
**feG and cardiovascular anaphylaxis**: Anaphylaxis induced by ovalbumin (OA) challenge in previously sensitized rats causes rapid reduction in blood pressure (control). feG treatment orally at the time of OA challenge dose-dependently inhibited anaphylaxis-induced hypotension. (mean ± sem, n = 5 to 6). Adapted from [38].

An early observation was that treatment with feG, like its predecessor SGP-T, inhibited the decrease in blood pressure associated with anaphylactic shock [38]. Challenge of sensitized rats with OA administered orally evoked a rapid drop in ventricular peak systolic pressure (VPSP) of 50 to 70 mm Hg. In normal rats or in unchallenged OA sensitized rats intravenous administration of SGP-T, FEG, or feG had no effect on resting VPSP at any dosage. However, in OA challenged rats, intravenous administration of each of the peptides 10 minutes prior to challenge significantly protected against the drop in VPSP compared to saline treated controls. Importantly, oral administration of feG 20 minutes before OA challenge also produced a dose-dependent inhibition of cardiovascular shock (Figure 4).

#### Leukocyte migration

**Figure 5:**
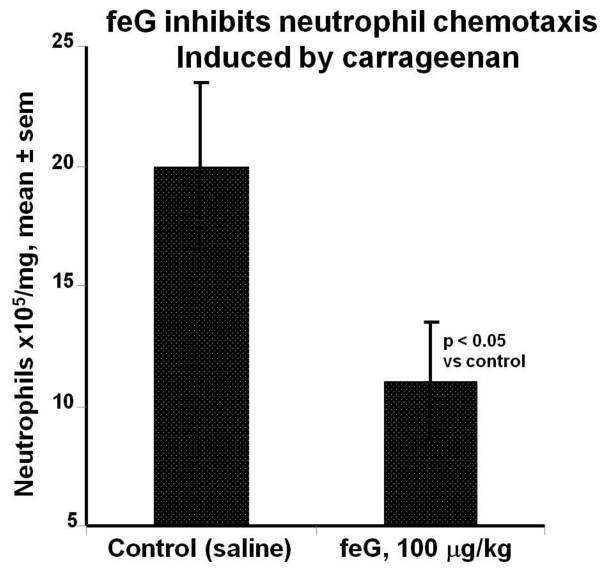
**feG and neutrophil migration**: Neutrophils migrate into carrageenan-soaked surgical sponges implanted subcutaneously in rats. feG, at a dosage of 100 μg/kg injected intraperitoneally at the time of sponge implantation, significantly inhibited neutrophil migration measured 24 hours after implantation. (mean ± sem, n = 6 to 10). Adapted from [39].

Neutrophil migration into carrageenan-soaked sponges 24 hours after subcutaneous implantation in rats was inhibited by intraperitoneal injection of feG at 100 μg/kg [39] (Figure 5). Neutrophil inflitration was significantly reduced by feG treatment in an acute pancreatitis model in mice [40] and also in a spinal cord injury model in rats [41].

**Figure 6:**
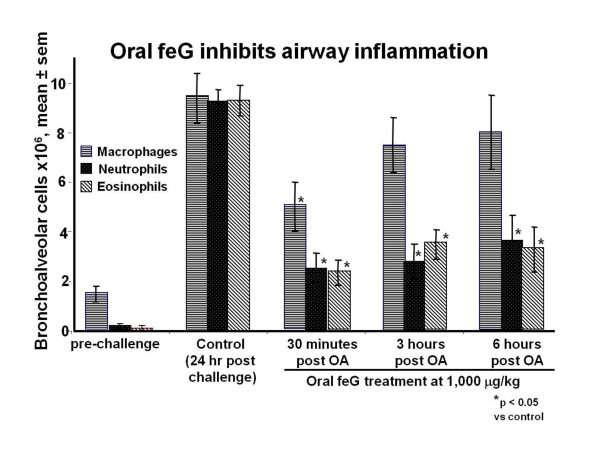
**Allergen induced by aerosol challenge with ovalbumin (OA) in previously sensitized rats causes pulmonary airway inflammation**: feG treatment orally 30 minutes, 3 hours, or 6 hours after OA challenge inhibited the influx of eosinophils and neutrophils into airways. Adapted from [44].

Oral challenge in OA sensitized rats induces systemic effects including increased circulating leukocytes, leukocyte infiltration into the heart, increased vascular permeability, and pulmonary inflammation [42]. Changes in vascular permeability occurred within 30 minutes, peripheral blood neutrophilia appeared by 3 hours, and significant accumulation of neutrophils in the heart, detected by a 75% increase in myeloperoxidase (MPO) content, was seen at 24 hours after oral OA challenge. Treatment with feG intraperitoneally 20 minutes before antigen challenge significantly inhibited the increase in vascular permeability, circulating leukocytes and neutrophils, and neutrophil infiltration into the heart. Intraperitoneal injection of feG at 100 μg/kg at the time of oral OA challenge of sensitized rats almost completely inhibited the increase in circulating neutrophils detected 18 hours after challenge [43]. Pulmonary airway inflammation in OA sensitized rats was also inhibited by feG. Oral treatment with feG 30 minutes to 6 hours after oral OA challenge significantly inhibited neutrophil and eosinophil numbers in airways 24 hours after challenge [44] (Figure 6). In another study, oral treatment with feG at dosages of 250 and 1,000 μg/kg 30 minutes before OA challenge inhibited influx of neutrophils, monocytes, and eosinophils into bronchoalveolar lavage fluid (BAL) but had no effect on lymphocytes [45].

**Figure 7:**
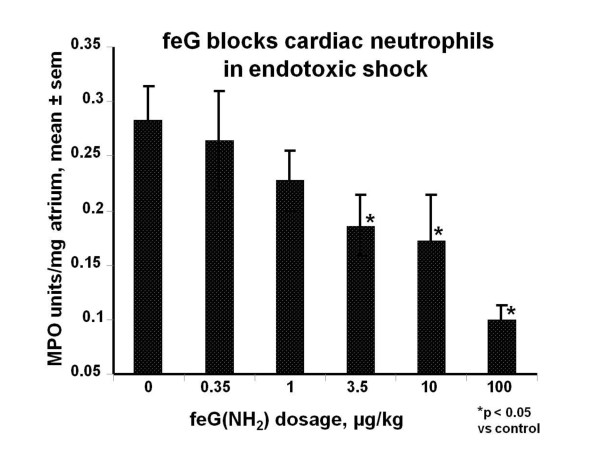
**Neutrophil accumulation in heart tissue**: Intravenous administration of lipopolysaccharide (LPS) in rats causes accumulation of neutrophils in heart tissue as detected by myeloperoxidase (MPO) activity in atrial slices 24 hours after LPS infusion. Intravenous treatment with a carboxamide derivative, feG(NH_2_), at the time of LPS infusion, dose-dependently inhibited MPO in atrial slices. (mean ± sem, n = 4 to 8). Adapted from [46].

Infusion of LPS in rats also causes accumulation of neutrophils in heart tissue in addition to acute hypotension [46]. Intravenous treatment with a carboxamide derivative, feG(NH_2_), at the time of LPS infusion, dose-dependently inhibited accumulation of neutrophils in atrial slices 24 hours after intravenous LPS (Figure 7). Orally administered feG (100 μg/kg) also significantly reduced the number of macrophages and neutrophils recovered in peritoneal lavage fluid 24 hours after LPS challenge [47].

#### Intestinal effects

**Figure 8:**
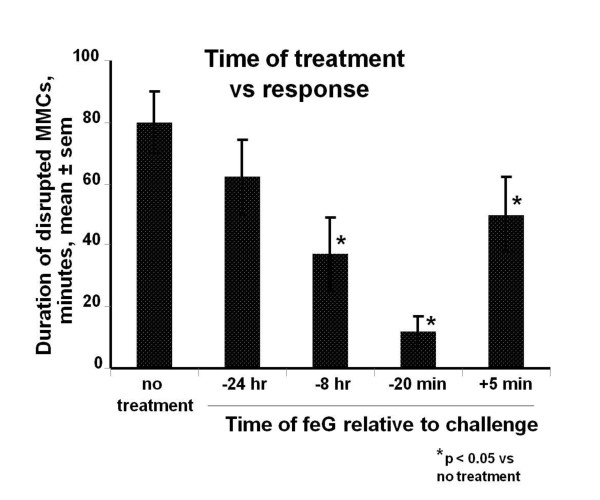
**feG and intestinal allergic responses**: Oral challenge with ovalbumin (OA) in sensitized rats results in disrupted intrinsic rhythmicity of migrating myoelectric complexes (MMC) in the small intestine. feG injected intravenously at 100 μg/kg up to 8 hours before challenge significantly reduced disruption in MMCs, suggesting a long biological half life (mean ± sem, n = 4 to 8). Adapted from [49]

Oral challenge with OA in sensitized rats also results in disrupted intrinsic rhythmicity MMCs in the small intestine, and in diarrhea in 85% of challenged animals [38,48]. Oral dosage of feG at 350 μg/kg at the time of OA challenge totally abolished the intestinal anaphylactic reaction and diarrhea in all rats tested. In a similar study feG given orally 30 minutes before OA challenge dose dependently inhibited anaphylaxis-induced intestinal motility, with maximal inhibition achieved at the highest dosage-100 μg/kg [49]. Interestingly, feG dosage (100 μg/kg) up to 8 hours before challenge afforded significant protection against intestinal anaphylaxis, suggesting a long biological half life (Figure 8) [49].

Infusion of LPS in rats also has acute effects on the intestine by disrupting the standard MMCs and produces a pattern of intense, irregular myoelectricity [50] Intravenous injection of feG 20 minutes before LPS dose-dependently reduced the length of time of disruption of jejunal MMCs. Interestingly, the carboxamide derivative, feG(NH_2_), was found to be more potent than feG in this endotoxemia model. feG given orally 20 minutes before LPS challenge inhibited disruption of MMCs in a bell shaped, dose-dependent manner, with 65 μg/kg providing maximal inhibition.

#### Effects on pulmonary inflammation and function

Effects of feG treatment were further studied in pulmonary inflammation models in rats sensitized with either OA or with larvae of *Nippostrongylis braziliensis *(Nb) and challenged by aerosol administration of the sensitizing antigens [45], Oral treatment with feG at 1 mg/kg 30 minutes prior to OA challenge significantly reduced airway hyper-responsiveness to methacholine measured 24 hours after challenge. In Nb sensitized rats feG significantly reduced tracheal smooth muscle contraction in response to aerosol Nb challenge.

**Figure 9:**
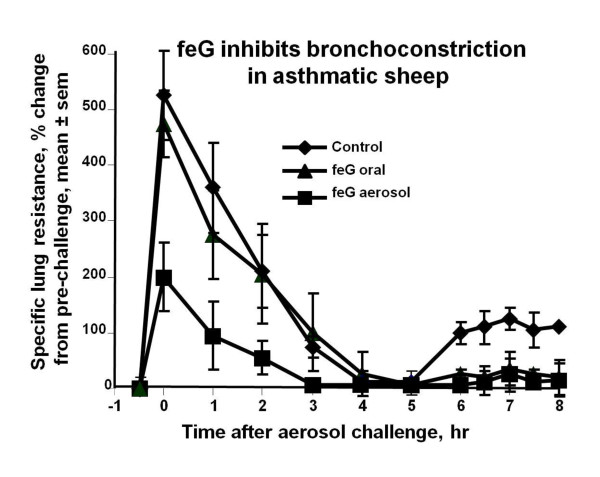
**feG and asthma in sheep**: In asthmatic sheep naturally sensitized to *Ascaris suum*, bronchoconstriction determined by measuring specific lung resistance (SR_L_) increased rapidly immediately after aerosol challenge, decreased to baseline values over 4 hours, but was followed by a secondary increase in SR_L _5 after hours post challenge. Inhaled feG at a dose of 30 mg/sheep reduced early as well as late increases in SR_L_, whereas treatment with feG intravenously (1 mg/kg) or orally (2 mg/kg) inhibited only late phase bronchoconstriction. (mean ± sem, n = 4 to 8). Adapted from [51].

In asthmatic sheep naturally sensitized to *Ascaris suum*, bronchoconstriction, determined by measuring specific lung resistance (SRL), and airway hyper-responsiveness to carbachol were measured in instrumented sheep after aerosol challenge with the antigen [51]. Bronchoconstriction (SRL) increased rapidly up to 500% immediately after aerosol challenge, decreased to baseline values over 3 hours, but was followed by a secondary increase in SRL 5 hours after challenge. Treatment with feG intravenously (1 mg/kg) or orally (2 mg/kg) had no effect on the early, acute phase increase in SRL, but inhibited the late phase increase by 72% and 78% respectively relative to challenged untreated controls (Figure 9). Inhaled feG, at a dose of 30 mg/sheep, reduced early (by 83%) as well late (by 88%) bronchoconstriction. Airway hyper-responsiveness to carbachol, measured 24 hours after antigen challenge, was significantly inhibited by pre-challenge treatment with feG intravenously, orally, or by aerosol delivery.

In cats sensitized to Bermuda grass allergen, administration of feG orally at 1 mg/kg immediately prior to allergen challenge resulted in a significant reduction in accumulation of eosinophils in bronchoalveolar lavage fluid [52]. However, daily treatment for 2 weeks in experimentally asthmatic cats had no measurable effect on airway inflammation [53]. This latter result suggests that further studies will be necessary to evaluate dosing regimens and formulation for feG (see Pharmacodynamic/pharmacokinetic considerations below).

### Vascular Permeability

The effects of feG on vascular permeability induced by antigen challenge and histamine have been studied in both rats and dogs. With both species intradermal injection of feG (10^-6^M to 10^-9^M) significantly reduced the increase in vascular leak of a dye (Evans blue) provoked by both active cutaneous anaphylaxis and histamine by up to 40% at high doses to ~20% at lower doses (unpublished observations).

### Other disease models: acute pancreatitis, spinal cord injury

**Figure 10:**
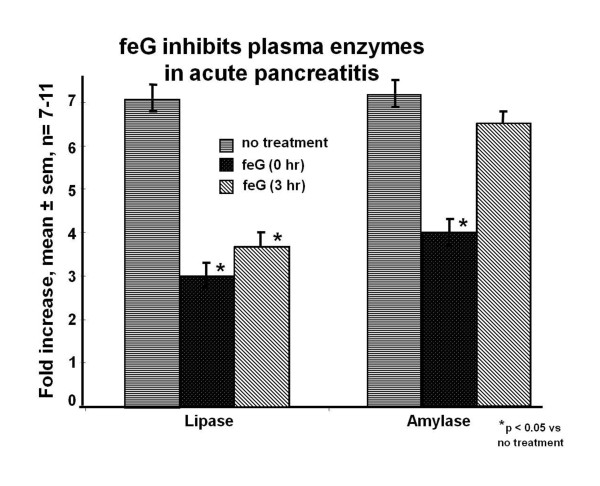
**feG and acute pancreatitis**. In acute pancreatitis, induced in mice by 12 hourly injections of caerulein, a single intraperitoneal dose of feG (100 μg/kg) administered at start of caerulein induction or 3 hours after start of induction, inhibited plasma lipase and amylase activity. Adapted from [40].

In acute pancreatitis, induced in mice by 12 hourly injections of caerulein, a single dose of feG (100 μg/kg) was administered intraperitoneally at induction (prophylactic) or 3 hours post induction (therapeutic) [40]. Plasma lipase activity was reduced in feG groups treated both prophylactically and therapeutically; amylase was reduced in feG groups treated prophylactically (Figure 10). Histologically, feG treatment reduced pancreatitis-induced edema and acinar cell necrosis.

**Figure 11:**
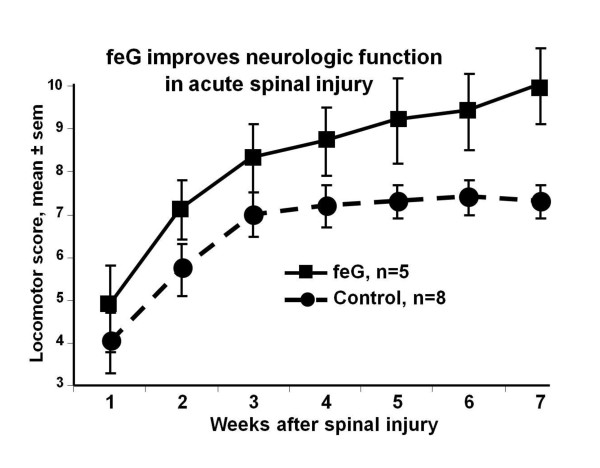
**feG and spinal cord injury**: In a spinal cord injury model induced by 60 second clip compression of the spinal cord, rats given feG intravenously at 200 μg/kg twice daily for 5 days had higher BBB locomotor scores compared to controls (p = 0.043) over 7 weeks following cord injury. Adapted from [54].

In a clip compression model of spinal cord injury in rats, leukocyte infiltration, free radical formation, and oxidative damage at the lesion site were quantified [41]. Neutrophil infiltration, detected by MPO activity, and activated phagocytic macrophages, identified by ED-1 expression, were present within 24 hours of injury. Intravenous feG treatment 2, 6, or 12 hours after injury reduced MPO activity, ED-1 expression, oxidative enzymes, free radical production, lipid peroxidation, and cell death (caspase-3 expression) in injured cord lesion sites. These anti-inflammatory and anti-oxidative actions of feG treatment correlated with improved neurological outcomes after spinal cord injury. In a similar spinal cord injury model feG given intravenously at 200 μg/kg twice daily for 5 days improved locomotor and allodynia scores relative to controls over 7 weeks following cord injury [54] (Figure 11).

### Pharmacodynamic/pharmacokinetic considerations

From a pharmacodynamic perspective, it appears that feG has a long biological half life. Single intravenous dosages of feG inhibit endotoxin-provoked accumulation of neutrophils in cardiac tissue for at least 24 hours [46] (see Figure 7). Single oral dosage of feG in OA sensitized challenged rats also inhibits neutrophil and eosinophil migration into airways for at least 24 hours [44] (see Figure 6). Likewise in asthmatic sheep intravenous, oral, or aerosol administration of feG blocks airway responsiveness for at least 24 hours after antigen challenge [51].

**Figure 12:**
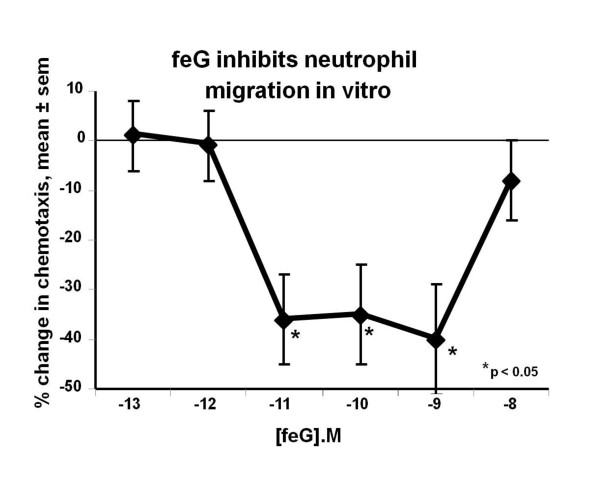
**feG and human neutrophils**: Incubation of human neutrophils with feG within a window of molar concentrations between 10^-11 ^to 10^-9 ^M downregulated platelet activating factor-(PAF) induced neutrophil migration *in vitro*. (mean ± sem, n = 3 to 7). Adapted from [39].

Bell shaped dose-response relationships were observed in various assays, so frequently as to not be dismissible as coincidental. First observed with SGP-T inhibition of anaphylaxis-induced hypotension in rats [55] and inhibition of neutrophil migration into carrageenan soaked sponges [22] (see Figure 5), feG treatment also resulted in a biphasic dose-response curve in an intestinal endotoxemia model [38]. *In vitro *incubation of human neutrophils with feG within a window of molar concentrations between 10^-11 ^to 10^-9 ^M down regulated platelet activating factor- (PAF) induced expression of CD 11b (AlphaM integrin chain) and PAF-induced neutrophil migration [39] (Figure 12). Within these same molar concentrations feG inhibited fibrinogen and fibronectin binding of peritoneal leukocytes from rats that had been infused with LPS 18 hours earlier. Binding of leukocytes from LPS treated rats to atrial slices was inhibited by feG *in vitro *at concentrations of 10^-9^M but not 10^-7^M [46]. These findings suggest that dosage of feG may be critical to achieve the desired therapeutic effect.

Pharmacokinetic studies, to our knowledge, have not been performed on feG in any species. However, results of preliminary pharmacokinetic and toxicokinetic studies have been performed on a closely-related salivary tripeptide (D-cyclohexylalanine-D-glutamate-glycine; (cha)eG) in rats, dogs, and monkeys (proprietary, in-house data, 2010). In rats and dogs oral dosages of 2,500 μg/kg of (cha)eG were required to achieve detectable plasma concentrations (>5 ng/mL). Oral bioavailability was estimated to be less than 1% in the rat. In monkeys detectable plasma levels of (cha)eG persisted for 24 hours following a single intravenous dosage of 10 mg/kg, with an apparent terminal half life of approximately 9 hours, consistent with pharmacodynamic findings in rats (see Figure 8). However, noting that in vitro feG is active within a window of concentrations of about 0.0035 to 0.35 ng/mL, and that in model studies in rats feG dosage of 100 μg/kg was consistently found to be effective regardless of route of administration, it must be concluded that the systemic bioactivity of feG occurs at concentrations well below minimum detectable plasma concentrations of current assays. In other words, the dosage riddle is unlikely to be solved by pharmacokinetics.

### Mechanism studies: Effect of feG on neutrophil chemotaxis, adhesion, and function

Results of *in vivo *studies point to the neutrophil as the primary target cell for the immunopharmacologic actions of feG and other bioactive factors produced by the salivary gland. Early results showed that SGP-T treatment inhibited neutrophil chemotaxis [22] as well as rolling [23].

#### Effect on adhesion

In peritoneal neutrophils collected from OA sensitized rats 24 hours after challenge, pre-treatment with feG had no effect on expression of the alpha integrin CD 11b but down regulated expression of the beta 1 integrin CD49 d (Alpha-4 integrin chain) [42]. *In vitro *incubation of human neutrophils with feG inhibited PAF induced neutrophil migration (see Figure 12) as well as expression of CD 11b [39]. In normal (unstimulated) neutrophils feG had no effect on neutrophil adhesion to gelatin, whereas in PAF-activated cells feG at 10^-11 ^and 10^-10^M significantly inhibited adhesion of human neutrophils. However, within molar concentrations of 10^-11 ^to 10^-9 ^M, feG had no effect on PAF-stimulated superoxide release or on phagocytotic activity, suggesting that feG modulates primarily neutrophil adhesion and migratory responses. Peritoneal neutrophils from OA sensitized rats 24 hours after challenge were also tested for expression of CD11b and CD16b (Fc-gamma RIIIb: Low affinity immunoglobulin gamma Fc region receptor IIIB). feG treatment (100 μg/kg orally) inhibited CD 11b antibody binding to peritoneal neutrophils in unchallenged but not in OA challenged rats. CD 16b binding, however, was inhibited by feG treatment in both challenged and unchallenged rats. *In vitro *(microtiter plates) feG inhibits adhesion of rat peritoneal leukocytes, but only if the cells were stimulated with PAF[43], indicating that feG's actions require cell activation. feG treatment also completely blocked the expression of the beta 1-integrin CD49 d on circulating neutrophils which was up regulated by OA challenge, but had no effect on CD11b expression. These and other findings led to the conclusion, that when administered *in vivo *feG prevents inflammation-induced reduction in cell adhesion as well as restoring its inhibitory effect *in vitro*.

#### Effect on oxidative activity

**Figure 13:**
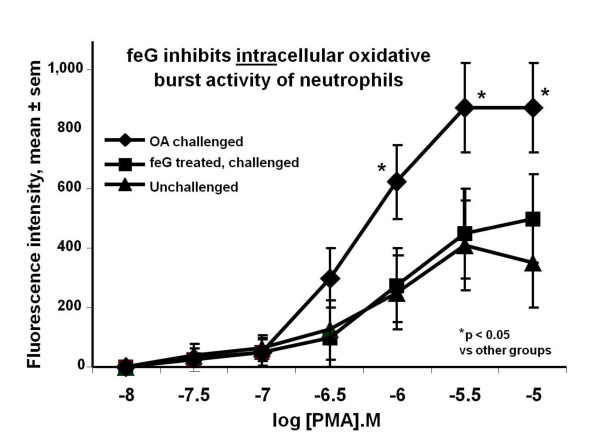
**feG and the oxidative burst**: - Dose-response for phorbol myristate acetate- (PMA) stimulated intracellular oxidative activity of circulating neutrophils 18 hours after ovalbumin (OA) challenge in OA sensitized rats. feG was injected intraperitoneally at 100 μg/kg at the time of challenge. Oxidative activity was measured using flow cytometry for a marker of oxygen free radicals, 123-dihydrorhodamine. (mean ± sem, n = 6 to 7). Adapted from [57].

Neutrophils, which play a key role in the development and perpetuation SIRS, inactivate and destroy virulent pathogens through the release of superoxide and enzymes and by phagocytosis [56]. In OA sensitized rats the extracellular release of superoxide anion by circulating neutrophils 18 hours after OA challenge was not modified by either OA challenge or feG treatment [57], confirming similar findings in previous studies [39]. However, incubation of the cells with phorbol myristate acetate (PMA), a protein kinase C (PKC) activator, increased intracellular release of reactive oxygen species as determined by flow cytometry for a marker of oxygen free radicals, 123-dihydrorhodamine. feG treatment at the time of challenge inhibited intracellular superoxide production by PMA-stimulated blood neutrophils 18 hours after challenge (Figure 13). These findings led to the speculation that feG reduces the capacity of neutrophils to generate reactive oxygen species by preventing the deregulation of PKC consequent to an allergic reaction.

Saliva, in addition to its role as a digestive aid, contributes significantly to lubrication, protection, defence and wound healing in the mouth. The importance of salivary glands and their secretions are poorly appreciated, and they are only taken seriously when salivary gland dysfunction results in decreased saliva flow. In humans this dysfunction contributes to difficulties in tasting, eating, swallowing, and speaking, and results in sores of the soft tissues of the mouth and periodontal disease. These pathologies also manifest in human patients with a variety of systemic diseases including - Sjögren's syndrome, rheumatoid arthritis, juvenile idiopathic (rheumatoid) arthritis, systemic lupus erythematosus (an inflammatory connective tissue disease), systemic sclerosis (sceloderma), primary bilary cirrhosis (an autoimmune disease of the liver), sarcidosis (a multisystem granulomatous disorder), infections with human immunodeficiency virus, herpes virus, hepatitis C, ectodermal dysplasia, chronic pancreatitis and depression [58].

Nonetheless, it should be recognized that the relationship between salivary glands and systemic health is bidirectional. "Oral infection may represent a significant risk-factor for systemic diseases, and hence the control of oral disease is essential in the prevention and management of these systemic conditions" [59]. Chronic inflammatory periodontal diseases are among the most prevalent chronic infections in humans, and many investigators have established a significant, albeit modest, positive association between periodontal disease and cardiovascular disease, which includes atherosclerosis, myocardial infarction and stroke. In addition, epidemiological associations have been made between periodontal diseases and chronic diseases such as diabetes, respiratory diseases and osteoporosis [60].

Likewise in veterinary medicine epidemiologic studies reveal that oral disease is the most common disease in all age groups of dogs and cats [61]. Moreover, there is evidence that oral infection also has systemic effects including renal, hepatic, pulmonary, and cardiac diseases; osteoporosis, adverse pregnancy effects, and diabetes mellitus [62], and can lead to systemic inflammation [63]. The severity of periodontal disease was found to be positively correlated with histological changes in kidneys, myocardium, and liver [64].

In this review we focused on SGP-T and its derivatives namely FEG and its D-isomeric derivative feG, which in themselves demonstrate the significant physiological and immunological modulation exerted by salivary gland peptides. These peptides have significant anti-inflammatory actions, as shown in animal models of endotoxic shock (Figures 1 &7), allergic and anaphylactic reactions (Figures 4, 6, 8 &9), pancreatic (Figure 10) and spinal cord injury (Figure 11).

feG, and its analogues, exhibit a distinctly different mechanism of anti-inflammatory action from corticosteroids and nonsteroidal anti-inflammatory drugs (NSAIDs). NSAIDs and corticosteroids have become the mainstay of anti-inflammatory agents in human and veterinary medicine. NSAIDs are popular owing to their immune sparing effect, especially since the discovery that they act by inhibiting cyclooxygenase (COX), an enzyme that catalyses the arachidonic acid cascade resulting in production of pro-inflammatory eicosanoids [65]. In contrast to enzymatic blockade, the tripeptide feG has multimodal activity and acts directly on activated leukocytes, specifically down regulating expression of integrins, thereby inhibiting chemotaxis (Figures 2 &12) and cell migration (Figure 5). Furthermore, feG inhibits the function of neutrophils by specifically inhibiting intracellular superoxide production by activated neutrophils (Figure 13), probably as a consequence of interruption of the signaling cascade that induces superoxide generation [66].

Hence feG and its analogues appear to represent a new class of anti-inflammatory agents which act on immune cells, the central regulators of all inflammation. The term "Immune Selective Anti-Inflammatory Derivatives" (ImSAIDs) is proposed for salivary-derived peptides to distinguish this class of agents from corticosteroids and NSAIDs. A closely-related salivary tripeptide ((cha)eG) is currently under investigation as an anti-asthmatic therapeutic in humans.

## Conclusions

Based on its mechanism of action and demonstrable *in vivo *pharmacologic activity, feG deserves evaluation in a number of situations characterized by over-exuberant or chronic inflammatory responses of human and veterinary significance associated with several major organ systems:

• Whole body and circulatory: sepsis, endotoxemia, SIRS [67];

• Gastrointestinal: pancreatitis, hepatitis, gastroenteritis, enteritis;

• Oral cavity: stomatitis

• Respiratory: asthma, acute pulmonary inflammation of diverse etiologies;

• Musculo-Skeletal: fibromyalgia, rheumatoid arthritis, equine laminitis (now characterized as a neutrophil-mediated inflammatory disease [68]);

• Nervous: spinal cord injury, peripheral nerve injury;

• Urinary tract: cystitis

Aside from these therapeutic potentials, feG may eventually prove to be useful as a vetraceutical or a nutraceutical [the term coined by Stephen DeFelice [69]] to reduce the incidence and severity of systemic and localized inflammations caused by intense exercise, poor oral health and other causes.

## List of Abbreviations

BAL: bronchoalveolar lavage fluid; CD11b: AlphaM integrin chain; CD16b: Fc-gamma RIIIb - Low affinity immunoglobulin gamma Fc region receptor IIIB; CD49 d: Alpha-4 integrin chain; (cha)eG: D-cyclohexylalanine-D-glutamate-glycine COX: cyclooxygenase; FEG: Phenylalanine-Glutamate-Glycine; feG: D-phenylalanine-D-glutamate-Glycine; IgE: immunoglobulin E; ImSAIDs: Immune Selective Anti-Inflammatory Derivatives; LPS: lipopoylsaccharide; MMC: migrating myoelectric complexes; MPO: myeloperoxidase;Nb: *Nippostrongylus brasiliensis; *NSAID: non steroidal anti-inflammatory drugs; OA: ovalbumin; PAF: platelet activating factor; PKC: protein kinase C; PMA: phorbol myristate acetate; SGP-T: submandibular peptide-T; SIRS: systemic inflammatory response syndrome; SRL: specific lung resistance; SMR1: submandibular rat-1; VCS-1: variable coding sequence-1; VPSP: ventricular peak systolic pressure

## Competing interests

DAG is a research veterinarian and a minority shareholder in a company which has commercial rights to salivary-derived peptides for veterinary use. RM and JSD have shares in a privately held company that is developing peptides and their analogues for therapeutic use.

## Authors' contributions

DAG conducted the literature search, wrote the first draft of the manuscript, and composed and edited the figures. RM contributed literature searches and the rewriting and editing. JSD and ADB provided important discussion and editorial comments. All authors read and approved the final manuscript.
